# Longitudinal point-of-care ultrasound training program for emergency medicine faculty in Oman: a Kirkpatrick model approach

**DOI:** 10.1186/s12909-025-07954-6

**Published:** 2025-10-02

**Authors:** Ashraf Elshehry, Saif Al Ghafri, Mahmood Al Jufaili

**Affiliations:** 1 Sultan Qaboos University Hospital, Muscat, Oman; 2 Sultan Qaboos University, Muscat, Oman

**Keywords:** Point-of-Care Ultrasound, Faculty Development, Emergency Medicine, Certification, Competency-Based Education, Program Evaluation, Kirkpatrick Model

## Abstract

**Background:**

Point-of-care ultrasound (POCUS) is an essential skill in emergency medicine (EM); however, gaps in faculty POCUS credentialing hinder effective trainee supervision and patient care. This study aimed to develop and evaluate a structured 12-month POCUS training program for EM faculty at Sultan Qaboos University Hospital (SQUH), Oman, with the primary goal of achieving independent practitioner certification (POCUS-IP) through the Oman Medical Specialty Board (OMSB).

**Methods:**

A prospective 12-month faculty development program was implemented, enrolling 10 EM faculty members. The intervention included a three-day POCUS bootcamp, supervised scanning across seven core ultrasound modules, written and practical examinations, and quarterly skill-maintenance sessions. Primary outcomes included the POCUS-IP certification rate, improvement in knowledge (pre- vs. post-test scores), and changes in POCUS utilization behaviors. Program outcomes were evaluated using the Kirkpatrick model, examining participant reaction (satisfaction), learning (knowledge and skills acquisition), behavior (changes in clinical practice and teaching), and results (rate of certification and educational impact on trainees).

**Results:**

All participants (100%) achieved OMSB POCUS-IP certification. Mean written test scores improved from about 79% pre-training to 90% post-training (*p* < 0.0001). Focus group discussions revealed perceived increased faculty confidence in integrating POCUS into clinical decision-making and more active teaching and supervision of trainees. The main barriers reported were a lack of time (60% of participants) and limited mentorship opportunities.

**Conclusion:**

A structured, longitudinal POCUS training program substantially improved faculty competency and achieved a 100% certification success rate. It highlights the educational value of structured POCUS training for faculty. However, further multicenter studies are warranted to assess long-term retention of skills and downstream effects on trainee performance and patient care.

**Supplementary Information:**

The online version contains supplementary material available at 10.1186/s12909-025-07954-6.

## Introduction

Point-of-care ultrasound (POCUS) has become an essential diagnostic and procedural tool in emergency medicine (EM), offering real-time insights that can enhance patient care [1]. Despite its recognized benefits, a significant gap exists in POCUS proficiency among EM faculty, particularly those who completed training before ultrasound was widely integrated into medical curricula. As of 2012, POCUS was identified as a core skill for graduating EM residents. However, many faculty who trained prior to 2008 lack formal POCUS credentials; in fact, half of EM residency programs in the United States report less than 50% of their faculty are credentialed to perform and teach POCUS [2].

This issue is pronounced at Sultan Qaboos University Hospital (SQUH) in Oman, where only 36% of EM faculty hold POCUS certification. This deficiency limits the faculty’s ability to supervise trainees effectively and may impede optimal patient care. Addressing this gap is imperative, as faculty competency in POCUS is crucial for modeling best practices and ensuring high-quality emergency care.

Recent literature underscores the need for structured faculty development in POCUS. For instance, targeted training interventions have been shown to bridge the competency gap among senior faculty, especially for those in predominantly cognitive specialties who have not regularly performed bedside procedures since residency [3].

This study aimed to develop and evaluate a structured 12-month POCUS training program for EM faculty at SQUH, with the primary goal of achieving OMSB certification as independent POCUS practitioners for all participants. We hypothesized that the program would significantly enhance faculty POCUS knowledge and skills, resulting in the successful certification of all participants. We also used the Kirkpatrick model as the evaluation framework to assess the program’s outcomes across multiple levels (Reaction, Learning, Behavior, and Results).

## Methods

### Study design and setting

This study was designed as a 12-month prospective interventional project (October 2022 – October 2023) to implement and evaluate a longitudinal POCUS training program for emergency medicine faculty. It was conducted in the Emergency Department of Sultan Qaboos University Hospital (SQUH) in Oman and received approval from the Institutional Medical Research and Ethics Committee. As an initial step, a needs assessment survey was administered to all EM faculty prior to the intervention. This survey evaluated faculty interest in POCUS, self-perceived competency levels, and preferences for maintaining POCUS skills, which helped inform the program’s content and structure. The overall goal was to create a program that met identified needs while adhering to national credentialing requirements for POCUS.

## Participants

All 22 emergency medicine faculty members at SQUH were invited to participate in the program. Faculty who already held POCUS Independent Practitioner (POCUS-IP) certification (*n* = 8) were not eligible, leaving 14 eligible faculty. Ultimately, 10 faculty members agreed and enrolled in the program (approximately 71% of those eligible), while four did not participate due to time constraints or lack of interest. All participating physicians were practicing EM faculty who had prior exposure to ultrasound: each had attended at least one POCUS training course within the past three years and utilized ultrasound in clinical practice, albeit infrequently. Thus, the cohort had a basic familiarity with POCUS but sought to improve and formalize their skills through the program.

### Intervention

#### Core POCUS bootcamp

 The intervention began with an intensive three-day “Core POCUS Bootcamp” in October 2022. This Bootcamp expanded on the standard two-day Oman Medical Specialty Board (OMSB) Core POCUS course by providing an extra day for skills practice. The first day of the Bootcamp included approximately 3 h of didactic lectures covering core ultrasound principles and applications, followed by about 4 h of hands-on scanning practice. The remaining two days were dedicated entirely to hands-on practice sessions. During these sessions, participants performed ultrasound examinations on simulated patients (trained volunteers acting as patients) under the direct supervision of POCUS instructors. All fundamental EM ultrasound applications were reviewed, reinforcing prior Learning and ensuring a common knowledge base among participants. This focused Bootcamp model is commonly used to rapidly upskill providers in POCUS and has been shown to improve knowledge and image acquisition skills in short courses [4]. Because all participants had recently completed introductory training, the Bootcamp placed heavy emphasis on extensive hands-on scanning to build confidence and proficiency in image acquisition. It also allowed each participant to perform many supervised scans in all core POCUS modules.

#### Supervised scan requirements

Participants were required to complete a log of supervised ultrasound scans for seven core POCUS applications, with minimum numbers as follows:

**Table:** 

POCUS Module	Number of supervised scans needed
Focused Assessment with Sonography for Trauma (FAST)	50 scan
Basic Echocardiography	25 scan
Lung for Pneumothorax and Pleural Effusion	15 scan
Abdominal Aortic Aneurysm (AAA) & Inferior Vena Cava(IVC)	25 scan
Gallbladder	25 scan
Renal for Hydronephrosis	25scan
Deep Vein Thrombosis (DVT)	25 scan

For each of these applications, the required scans had to be performed under the supervision of POCUS instructors as per OMSB certification criteria. These minimum numbers were set to ensure adequate repetition, reflecting evidence that competency in emergency ultrasound requires dozens of practice scans per modality.

All scan attempts were directly observed and verified by POCUS instructors. Supervision was provided by the Bootcamp course faculty as well as four additional SQUH EM faculty who were themselves certified POCUS-IP instructors (selected from the eight certified faculty in the department). This approach enhanced a “train-the-trainer” model, using experienced faculty to mentor their peers. The supervisors provided real-time feedback on imaging technique and interpretation, and signed off on completed studies once they deemed the images adequate. Participants progressed at their own pace but generally fulfilled the scan requirements for all seven modules within 3 months of the Bootcamp course.

#### Assessments and remediation

 Once participants completed the required supervised scans for all modules, they proceeded to formal assessments of knowledge and practical skills. The written assessment was a 40-question single-best-answer multiple-choice exam covering key concepts for all seven POCUS modules (e.g., image interpretation, anatomy, sonographic findings, and clinical integration). An identical 40-question test was administered before the program (pre-test) and after completion (post-test) to gauge knowledge improvement.

The practical assessment evaluated hands-on proficiency: participants were tested via live scanning of real patients for each POCUS application under the observation of expert examiners (POCUS instructors). During these practical exams, faculty had to demonstrate correct scanning techniques and accurately identify or interpret relevant findings in each module (for instance, obtaining cardiac views or detecting abdominal free fluid). According to OMSB rules, passing the written and practical components in all required modules is mandatory for POCUS-IP certification. Participants who did not initially pass one or more module assessments were offered targeted remediation. Specifically, if a faculty member struggled with a particular exam (notably the cardiac ECHO, gallbladder, or AAA modules), they received additional coaching and practice on that ultrasound application, followed by a repeat assessment within two weeks. This remediation process ensured that all participants ultimately reached the competency standard. Only after successfully passing all post-tests (including any necessary re-tests) were participants submitted for credentialing as independent POCUS practitioners.

#### Ongoing skill maintenance

 In addition to the initial training and assessments, the program incorporated structured maintenance of skills to promote retention. After achieving certification, faculty were required to participate in quarterly POCUS skill refresher sessions over the following year. Each session was a one-day, approximately 6-hour hands-on workshop in which the newly certified faculty scanned real patients under the supervision of POCUS instructors. These sessions served to reinforce techniques and prevent skill decay. Participation in at least one maintenance session per quarter was mandatory for all program graduates, in line with OMSB recommendations to ensure continued competency. This longitudinal support structure is consistent with best practices in medical education, emphasizing that initial training should be followed by opportunities for deliberate practice and feedback to solidify skills [4].

### Outcomes and evaluation

#### Evaluation framework

We evaluated program outcomes using Kirkpatrick’s four-level model of training evaluation. In this framework, Level 1 is the participants’ Reaction (satisfaction with the training), Level 2 is Learning (knowledge and skill acquisition), Level 3 is Behavior (changes in on-the-job Behavior, in this case POCUS usage and teaching), and Level 4 is Results (primarly, the certification rate and secondary, the broader impacts, such as effects on trainees or organizational outcomes). While direct trainee performance and patient outcomes were not measured, focus group feedback and qualitative comments were analyzed to capture perceived changes.

We defined several specific outcomes corresponding to these levels:

- POCUS-IP certification rate (Level 4 – Results): The primary outcome was the proportion of participants who successfully achieved POCUS Independent Practitioner certification through OMSB by the end of the program. Achieving certification required meeting all training requirements and passing all assessments. This outcome represented the program’s ultimate goal of producing independent POCUS practitioners and indicates the potential institutional impact (increasing the pool of certified faculty).
- Knowledge improvement (Level 2 – Learning): We assessed gains in ultrasound knowledge by comparing each participant’s score on the 40-question written test before vs. after the program. The difference between the post-test and pre-test scores (in percentage of questions correct) was calculated for each participant to quantify knowledge improvement. We expected significant score increases if the program effectively taught key POCUS concepts and protocols.
- Practical skill competency (Level 2 – Learning): Improvement in hands-on ultrasound skills was measured via the practical examinations. We recorded whether each participant successfully passed the final practical exam in all modules (after completing the supervised scan phase). We also noted any need for remediation attempts. Successful completion of all modules’ practical exams indicated attainment of the required skill level (ability to acquire and interpret ultrasound images) for independent practice.
- POCUS utilization and behavior change (Level 3 – Behavior): Changes in how participants utilized POCUS in their clinical practice and teaching were assessed through self-report and external observations. At program completion, we administered a survey asking participants to describe any changes in their use of ultrasound for patient care and in teaching or supervising trainees, compared to before the program. In addition, we conducted two focus group discussions (one with EM residents and one with POCUS-certified faculty instructors in the department) to gather independent perspectives. Focus group participants were asked whether they had noticed differences in the faculty’s ultrasound use or teaching behaviors since the program. This combination of self-reported and observer-reported data captured behavioral changes beyond what could be measured by exams (e.g., whether faculty were actually using POCUS more frequently at the bedside or engaging residents in ultrasound learning).
- Participant satisfaction (Level 1 – Reaction): Faculty satisfaction with the program was evaluated by an end-of-program survey. Participants rated their overall training experience (e.g., on a Likert scale from very dissatisfied to very satisfied) and indicated whether they would recommend the program to colleagues. The survey also included open-ended questions for feedback on the most valuable aspects of the program and suggestions for improvement. High satisfaction scores and positive feedback would indicate that the program was well-received and perceived as beneficial.

### Data collection and analysis

#### Quantitative data

 All participants completed the baseline 40-question written test (knowledge assessment) and the needs assessment survey before the intervention. After completing the training, participants repeated the written knowledge test and responded to a post-program survey (including questions on POCUS usage and satisfaction, as described above). The program tracked data on each participant’s progress (number of scans completed in each module, assessment results, etc.). The primary outcome of certification was recorded as a binary outcome (achieved vs. not achieved). Knowledge test scores were recorded as percentage correct for pre- and post-tests. The program’s effect on knowledge was analyzed by comparing the mean pre-test and post-test scores using a paired t-test (two-tailed) for matched samples. Each participant served as their own control in this comparison. We set the significance threshold at *p* < 0.01 (1% level) to account for the relatively small sample and multiple outcome measures, using a more stringent criterion for detecting improvement. A statistically significant increase in test scores would support that the intervention led to knowledge gains. We also calculated descriptive statistics (mean ± standard deviation) for the test scores and reported the absolute improvement in the score (percentage points). Other quantitative data, such as the proportion of participants changing their frequency of POCUS use (from the survey) or the 100% certification rate, were summarized as percentages. All statistical analyses were performed using standard software (e.g., SPSS), and given the small sample size (*n* = 10), no multivariable analyses were undertaken.

#### Qualitative data

 Qualitative feedback was obtained from the post-program surveys (which included open-ended comment questions) and the two focus group discussions. The focus groups were conducted approximately one month after program completion – one with 6 EM trainees and one with 4 POCUS-certified faculty instructors.

We employed a semi-structured focus group format with open-ended guiding questions to explore perceived changes in faculty POCUS behavior and competence after the program. For example, the resident focus group was asked whether they had observed any differences in how faculty use or teach ultrasound since the training (e.g., “Have you noticed any changes in how faculty use POCUS in patient care or teaching since the program?“). The instructor focus group was similarly prompted to discuss improvements in the participating faculty’s ultrasound skills, confidence, or teaching approach (e.g., “In what ways have the trained faculty’s POCUS skills or usage changed post-training?“). The facilitator took detailed notes and debriefed immediately to capture key points and verbatim quotes. Guiding questions included: [1] “Have you noticed any changes in how faculty use or teach POCUS since the program?” [2] “In what ways have the trained faculty’s POCUS skills or usage changed post-training?” [3] “Can you describe a recent example of integrating POCUS into patient care?” [4] “What barriers or facilitators have you experienced when using POCUS after the program?”

These notes and the written comments from the surveys constituted the qualitative dataset for analysis. We analyzed the qualitative data using thematic analysis, following standard approaches in medical education research [5].

Overall, by combining test score analysis, competency assessments, behavioral surveys, and thematic feedback analysis, we aimed to comprehensively evaluate the faculty POCUS development program’s effectiveness. This mixed-methods approach follows recommended practices in medical education research to assess multiple outcome levels (from knowledge and skills to Behavior and satisfaction) for faculty development initiatives.

## Results

Program outcomes are presented in alignment with the Kirkpatrick framework:

- Level 1 (Reaction): Participant satisfaction was very high. All 10 faculty (100%) rated the training as “satisfied” or “very satisfied” in the post-program survey, indicating a strongly positive reaction to the program.
- Level 2 (Learning): There were significant gains in knowledge and skills. Mean written test scores increased from 79.0% before training to 90.5% after training (*p* = 5.2 × 10⁻⁸), and all participants successfully passed the practical skills examination (after remediation for 3 participants who needed an extra attempt in one or two modules). These results demonstrate effective Learning of POCUS content and skills.
- Level 3 (Behavior): Participants reported, and observers confirmed, that faculty integrated POCUS into clinical practice more frequently after the program. Faculty felt more confident using ultrasound for diagnosis and teaching, and emergency medicine residents noted more frequent bedside ultrasound teaching and supervision by faculty. All participants maintained POCUS logbooks and contributed cases to a departmental ultrasound archive, providing evidence of sustained POCUS use in their day-to-day practice.
- Level 4 (Results): all 10 participants (100%) successfully fulfilled the OMSB requirements and achieved certification as POCUS Independent Practitioners. EM residents perceived improved training and learning opportunities as a result of having more POCUS-certified faculty; however, no direct measurements of trainee POCUS competency or patient care outcomes were collected in this study. The benefits at this highest level remain **perceived** and anecdotal; further research is needed to objectively confirm improvements in trainee performance or clinical outcomes resulting from the faculty development program.

### Participant characteristics

Of the 22 faculty members invited, 10 enrolled in the program, consisting of 4 junior and six senior faculty. All participants had prior informal exposure to POCUS, having completed at least one POCUS course within the last three years. However, their use of ultrasound in clinical practice was infrequent. The baseline needs assessment results revealed that 70% of participants reported limited prior POCUS exposure, and the mean self-rated confidence in performing and interpreting POCUS was 3.5/10, indicating a substantial need for structured training. The most commonly reported barrier to training was time constraints (60%), followed by limited access to structured mentorship opportunities. These findings confirmed a strong need for the structured training intervention.

### Primary outcome: certification rate

By the end of the program, all 10 participants (100%) successfully achieved POCUS Independent Practitioner (POCUS-IP) certification through the Oman Medical Specialty Board (OMSB). This outcome represents a significant institutional shift, increasing the proportion of certified emergency medicine faculty at SQUH from 36 to 82%, strengthening the department’s capacity for high-quality POCUS education and supervision [6].

### Knowledge improvement

**Fig. 1:**
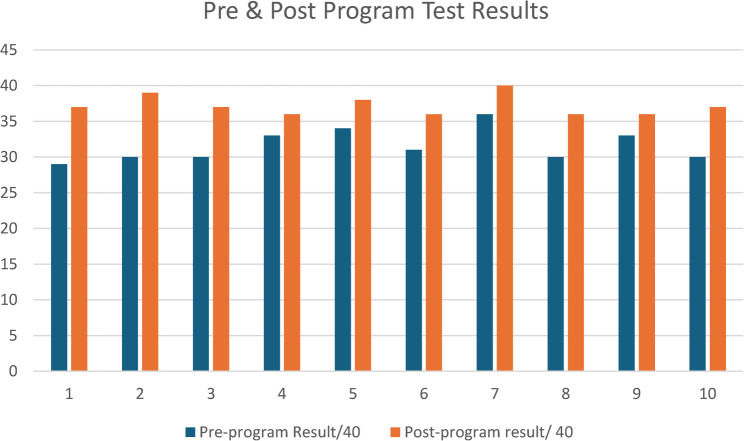
Comparison of participants’ written exam scores before and after POCUS training

A significant improvement in POCUS knowledge was observed among participants. The mean written exam score increased from 79.0% (± 5.8%) pre-training to 90.5% (± 4.0%) post-training, reflecting an average gain of 11.5% points. Statistical analysis using a paired t-test confirmed that this improvement was highly significant (*p* = 5.2 × 10⁻⁸), indicating a robust educational impact. The calculated effect size (Cohen’s d ≈ 1.8) suggests a strong learning effect among participants. Fig. 1 illustrates individual participant improvements in test scores.

### Practical competency assessment

All participants successfully passed the practical examinations across all seven required POCUS modules. However, three participants (30%) required one remediation attempt before passing one or more modules, specifically in the ECHO, gallbladder, and AAA assessments. After targeted remediation, all participants demonstrated competency in performing and interpreting ultrasound scans on real patients, meeting the OMSB certification standards [7]. This ensured that all certified faculty could perform POCUS applications on real patients at a level appropriate for independent practice. It also indicates that the combination of initial training and, when necessary, remediation was sufficient to bring each faculty member to competency.

### Behavioral changes in POCUS utilization

Post-program qualitative feedback from focus groups and surveys indicated a marked increase in faculty engagement with POCUS in clinical practice. Emergency medicine residents in the focus group described several noticeable changes in faculty behavior after the training. They observed that faculty were providing more frequent POCUS teaching during clinical shifts and consistently encouraging residents to perform ultrasound on appropriate cases. The residents also noted that faculty now more often incorporated ultrasound findings into their clinical decision-making, openly demonstrating how POCUS results informed patient management. These observations align with the faculty participants’ own self-reported behavior changes: the trained faculty felt greater confidence in using ultrasound for diagnostic decision-making and in supervising trainees. Departmental POCUS instructor faculty further corroborated these reports, noting that the newly certified faculty were integrating POCUS more regularly into bedside patient assessments and teaching activities with learners. The participating faculty also emphasized that the structured quarterly skill maintenance sessions were crucial in maintaining and reinforcing their skills [8]. During routine clinical shifts, newly certified faculty reported performing or supervising approximately 5–15 POCUS scans per 8-hour shift, depending on case mix and Emergency Department workload.

Moreover, each participating faculty member maintained a POCUS logbook to document their ultrasound scans throughout the program. These logbooks recorded all supervised scans performed during training and were continued by faculty after achieving certification, providing a quantitative record of ongoing ultrasound use. The department also created a centralized repository where faculty uploaded notable POCUS cases (such as positive diagnostic findings), thereby fostering knowledge sharing and reflection. The presence of these personal logbooks and the shared case archive offers **objective evidence** that faculty sustained active POCUS utilization in clinical practice beyond the training period.

### Participant satisfaction

Program satisfaction was overwhelmingly positive. In the post-program survey, 100% of participants rated their overall experience as “satisfied” or “very satisfied.” Faculty particularly appreciated the extensive hands-on training, the structured and progressive learning approach, and the ongoing skill reinforcement through the quarterly maintenance sessions. One faculty member stated, “I feel much more confident scanning critically ill patients now,” while another noted, “This program made it easier for me to teach our residents ultrasound.” Such feedback underscores the program’s impact on faculty skill acquisition and its downstream effects on trainee education [9].

## Discussion

### Summary of key findings

This study demonstrates that a structured, 12-month faculty POCUS development program can lead to 100% certification of participants and significant improvements in ultrasound knowledge and practical skills among emergency medicine (EM) faculty. The program effectively addressed existing gaps in faculty credentialing, increasing the proportion of certified EM faculty from 36 to 82% at Sultan Qaboos University Hospital (SQUH). Additionally, post-program surveys and focus group discussions confirmed increased faculty confidence in performing and teaching POCUS and greater integration of ultrasound into clinical decision-making. These findings reinforce the need for structured, longitudinal training approaches to enhance faculty competency in POCUS.

### Implications for medical education and clinical practice

This study’s high certification success rate has meaningful implications for medical education and patient care. Before the program, faculty lacked formal POCUS credentials, which limited their ability to supervise trainees effectively. This gap has been recognized in previous studies, where a lack of faculty credentialing resulted in inconsistent ultrasound teaching and a reliance on self-taught techniques. By certifying all participating faculty, the program ensured that EM residents now have a larger pool of qualified instructors for bedside ultrasound. Trainees can receive standardized POCUS instruction, direct faculty supervision during scans, and structured competency assessment – all of which are critical for a robust residency training experience [10].In essence, the program helped create a more supportive learning environment for ultrasound within the department.

Qualitative feedback from our residents supports these improvements: trainees reported that faculty were now providing more bedside POCUS teaching and consistently encouraging them to perform ultrasound during patient evaluations. Faculty not only increased their use of ultrasound in clinical care but also actively involved residents in interpreting the findings and making clinical decisions based on POCUS results. This suggests a richer learning experience for trainees and a stronger culture of ultrasound utilization in the department. Notably, residents in our program have structured 4-week ultrasound rotations in year 1 and year 2, during which formal supervised scans are preferentially signed off by designated POCUS instructors; outside these rotations, the newly certified faculty increasingly supervise or perform scans with residents during ED shifts when clinically appropriate.

It is important to note, however, that these downstream effects on trainees are based on observations andself-reportst rather than objective measurements. We did not assess resident POCUS competency or patient outcomes directly in this study. While the resident feedback is encouraging and suggests a positive educational impact, further research is necessary to determine whether improved faculty POCUS skills ultimately translate into significantly enhanced trainee performance or patient care outcomes.

### Comparison to existing literature

Our findings align with prior research on faculty POCUS training programs, yet offer notable enhancements. A study by Maw et al. (2016) evaluated an ultrasound training course for internists, reporting lower certification rates and a shorter program duration (10 weeks). In contrast, our 12-month curriculum with mandatory scanning quotas and skill maintenance sessions likely contributed to higher success rates. Similarly, the Dversdal et al. (2021) train-the-trainer model highlighted the importance of ongoing skill reinforcement, a principle integrated into our program through quarterly refresher sessions. This longitudinal approach may have been key in preventing skill decay, an issue observed in shorter, intensive POCUS courses (Blehar et al., 2015).

One of the most distinctive aspects of our program was the mandatory supervised scanning requirements. Unlike many faculty development initiatives that rely on self-directed scanning, participants in our study had to complete a predefined number of scans under supervision before advancing to certification assessments. This structured approach ensured sufficient hands-on practice, aligning with recommendations from professional organizations such as the American College of Emergency Physicians (ACEP) and the Society of Point-of-Care Ultrasound (SPOCUS) [11]. Incorporating real patient assessments during quarterly skill maintenance sessions is also a novel addition, reinforcing ongoing competency rather than a one-time skills acquisition.

### Challenges and barriers

Despite the program’s success, time constraints emerged as the most significant barrier to faculty participation, with 60% of faculty identifying this as a challenge. This is consistent with prior literature, where faculty training initiatives often compete with clinical and administrative duties, limiting participation [12]. Some participants struggled to balance ultrasound training with their routine patient care responsibilities, which may have delayed the completion of supervised scans. To address this, institutions may need to consider formal integration of POCUS faculty training into academic schedules, ensuring protected time for skill development [13]. Departments could also allocate dedicated ultrasound training days or reduce non-clinical burdens to facilitate faculty engagement. Another challenge was variability in prior ultrasound exposure. While all participants had attended at least one POCUS course in the past three years, their actual clinical use of ultrasound varied, potentially influencing individual learning curves. Future programs might benefit from pre-course competency assessments, allowing for targeted skill development based on baseline proficiency levels. Additionally, incorporating blended learning approaches, such as asynchronous online modules, could supplement hands-on training and address scheduling conflicts [14].

### Limitations and future directions

Several limitations of this study should be acknowledged. First, it was conducted at a single academic center with a relatively small number of participants (*n* = 10). This limits the generalizability of our findings to other settings. The context at our institution—including available resources, culture, and leadership support—may differ from elsewhere. Additionally, participation in our program was voluntary, introducing potential selection bias. The faculty who enrolled were likely those most motivated and interested in POCUS, perhaps contributing to the program’s high success rate. In other words, our results might represent a best-case scenario achieved with highly engaged faculty, and outcomes could be more modest with a less self-selected group.

Second, the study primarily focused on faculty certification and short-term competency outcomes. While achieving 100% certification is a significant milestone, we did not formally evaluate long-term retention of POCUS skills. Additionally, we did not collect objective data on how frequently faculty utilized POCUS in clinical practice or how they integrated ultrasound into patient care and teaching after the program. Therefore, the sustained impact on trainee education and patient outcomes remains uncertain. Further studies should investigate long-term skill retention and whether faculty-led POCUS instruction translates into measurable improvements in trainee education or patient outcomes [7]. Nonetheless, we have anecdotal evidence of continued POCUS use by the faculty beyond the program: all participants have maintained regular ultrasound practice, as demonstrated by ongoing entries in their POCUS logbooks and continued sharing of positive scan findings in a departmental archive. This suggests sustained engagement with ultrasound, but future studies should include long-term follow-up assessments — for example, re-testing faculty skills or surveying their POCUS usage 6–12 months or even several years after program completion — to evaluate the durability of competency. It would also be valuable to examine whether faculty who undergo training continue to mentor others and help spread ultrasound skills (a multiplier effect that we did not measure but could be a beneficial outcome).

Third, the program’s feasibility and resource requirements should be considered. Our faculty development initiative was facilitated by significant support: ultrasound machines were provided free of charge by collaborating companies for the training period, and departmental resources (including protected faculty time, simulation equipment, and administrative support) covered the remainder of the program needs. Not all institutions may have access to such support, which could limit the generalizability of this model. Another area for future exploration is the cost-effectiveness of longitudinal POCUS faculty development programs. While short, intensive workshops may be more feasible in some settings, their effectiveness in ensuring long-term competency remains questionable. Future studies could compare different training approaches to determine if shorter but high-yield interventions can achieve similar outcomes or if ongoing skill maintenance strategies are essential for sustained proficiency. In our context, ultrasound machines used for training were provided free of charge by collaborating companies, and remaining needs were supported by the department; consequently, a formal cost analysis was not performed, which may limit direct replication in settings without similar support.

Finally, evaluating the program through the lens of the Kirkpatrick model highlights both its strengths and areas for further inquiry. We demonstrated clear successes at Level 1 (Reaction) with uniformly positive participant satisfaction, Level 2 (Learning) with significant improvements in knowledge test scores and practical skill competency, and Level 3 (Behavior) with evidence of changes in clinical practice and teaching behavior post-training. However, at Level 4 (Results), our evidence was limited to perceptions and indirect indicators. Residents and peers perceived an enhanced learning environment for trainees, but we did not measure any concrete trainee outcomes or patient outcomes. Future studies on faculty POCUS development should strive to include Level 4 evaluations, such as testing whether resident cohorts taught by newly trained faculty perform better in ultrasound proficiency, or tracking patient care metrics (like diagnostic accuracy or procedural success rates) in settings where faculty have undergone POCUS training versus not. Such data would provide stronger evidence of the ultimate impact of faculty training on medical education and clinical practice.

## Conclusion

This study demonstrated that a structured, longitudinal POCUS faculty development program was highly effective, with all participating emergency medicine faculty (100%) achieving independent practitioner certification and substantial improvements in knowledge, skills, and confidence. The program’s design, combining an immersive bootcamp, supervised scanning with mentorship, formal assessments with remediation, and ongoing refresher sessions, enabled sustained skill acquisition while addressing common barriers such as time constraints.

Framed within the Kirkpatrick model, outcomes were strong at Levels 1–3, showing high participant satisfaction (Reaction), significant learning gains (Learning), and clear behavioral changes in clinical practice and teaching (Behavior). At Level 4, the achievement of universal faculty certification represents a meaningful institutional result. However, other downstream outcomes, including measurable effects on resident performance and patient care, were not assessed and remain an important area for future study.

Future multicenter research is recommended to validate these findings, evaluate long-term retention of faculty POCUS skills, and determine whether enhanced faculty proficiency translates into measurable improvements in residency training and patient care outcomes.

## Supplementary Information

Supplementary Material 1.

## Data Availability

The de-identified datasets generated and analyzed during the current study are available from the corresponding author on reasonable request.

## Abbreviations

AAA: Abdominal Aortic Aneurysm
ACEP: American College of Emergency Physicians
DVT: Deep Vein Thrombosis
ECHO: Echocardiography
EM: Emergency Medicine
FAST: Focused Assessment with Sonography for Trauma
IVC: Inferior Vena Cava
OMSB: Oman Medical Specialty Board
POCUS: Point-of-Care Ultrasound
POCUS-IP: Point-of-Care Ultrasound Independent Practitioner
SPOCUS: Society of Point-of-Care Ultrasound
SQUH: Sultan Qaboos University Hospital
