# Supplementary material for: Longitudinal point-of-care ultrasound training program for emergency medicine faculty in Oman: a Kirkpatrick model approach
Source: BMC Med Educ. 2025 Oct 2;25:1346. doi: 10.1186/s12909-025-07954-6 (PMC12492678; doi:10.1186/s12909-025-07954-6)
Supplement: Supplementary file 1 — Supplementary Material 1. [file 12909_2025_7954_MOESM1_ESM.pdf]

POCUS development program for emergency medicine faculty in SQUH

Questionnaire

* Indicates required question

1. What is your grade? *

   Mark only one oval.

   - SMO
   - Specialist
   - Senior Specialist
   - Consultant
   - Senior Consultant

2. POCUS is a core competency every emergency physician should be skilled in; *

   Mark only one oval.

   - Totally disagree
   - Disagree
   - Neutral
   - Agree
   - Totally agree

3. When was the last time you attended a POCUS course (as a student or instructor)? *

   Mark only one oval.

   - This year
   - Last year
   - 2 years ago
   - 3 -5 years ago
   - 5-10 years ago

4. When was the last time you practiced POCUS on a real patient? *

   Mark only one oval.

   - In my last duty this week
   - Last week
   - Last month
   - I can't remember

5. I'm practicing POCUS during my duty. *

   Mark only one oval.

   - Never
   - Rarely
   - Sometimes
   - Often
   - Always

6. Regarding acquiring and interpreting e-FAST POCUS *

   Mark only one oval.

   - I need more training
   - I need supervision
   - I need training and supervision
   - I do it independently

7. Regarding acquiring and interpreting AAA POCUS *

   Mark only one oval.

   - I need more training
   - I need supervision
   - I need training and supervision
   - I do it independently

8. Regarding acquiring and interpreting DVT POCUS *

Mark only one oval.

- I need more training
- I need supervision
- I need training and supervision
- I do it independently

9. Regarding acquiring and interpreting renal POCUS *

Mark only one oval.

- I need more training
- I need supervision
- I need training and supervision
- I do it independently

10. Regarding acquiring and interpreting hepato-biliary POCUS *

Mark only one oval.

- I need more training
- I need supervision
- I need training and supervision
- I do it independently

11. Regarding acquiring and interpreting Basic Echocardiography. *

Mark only one oval.

- I need more training
- I need supervision
- I need training and supervision
- I do it independently

12. How do you see the role of POCUS in our department? *

Mark only one oval.

- Limited role
- Neutral role
- Active role
- Highly active role

13. How do you see the POCUS practice in our department? *

Mark only one oval.

- Individual direction
- Departmental direction
- Duty in-charge direction

14. I am satisfied with my POCUS skill and practice in the department *

Mark only one oval.

- Strongly disagree
- Disagree
- Neutral
- Agree
- Strongly agree

15. I feel confident when a colleague, trainee, GFP or intern ask me to teach or supervise him/her in doing basic POCUS? *

Mark only one oval.

- Not that much
- Neutral
- Confident in some modules
- Confident in all the basic modules mentioned above

16. What do you think we need in our department to improve POCUS skill among us? *

Mark only one oval.

- More pracice
- Regular Practice and teach
- Refresher course to maintain the skill
- Structured training and quality assurance measures

17. Are you interested to participate in a POCUS training program in the department to improve and maintain your POCUS skill? *

Mark only one oval.

- Interested
- Not interested

This content is neither created nor endorsed by Google.

Google Forms
